# The life cycle of the tapeworm Dibothriocephalus latus in the Alpine region: What we know, what we miss, and how it is maintained

**DOI:** 10.1016/j.ijppaw.2026.101294

**Published:** 2026-09-29

**Authors:** Viktor Kovalov, Barbora Trubenová

**Affiliations:** Department of Aquatic Ecology, Swiss Federal Institute of Aquatic Science and Technology (Eawag), Ueberlandstrasse 133, Dübendorf, 8600, Switzerland; Institute of Integrative Biology, D-USYS, ETH Zurich, Universitätstrasse 16, Zürich, 8092, Switzerland

**Keywords:** Cestoda, Diphyllobothriosis, Parasite ecology, Alpine region, Triploidy, Wastewater treatment

## Abstract

Dibothriocephalus latus is a broad fish tapeworm with a complex life cycle that requires three host stages — copepods, fish, and mammals, including humans. Over the last 25 years, the prevalence of *D. latus* has noticeably declined in many parts of Europe, with the exception of the Alpine region. Recent studies have shown that *D. latus* is prevalent in fish from sub-alpine lakes, the second intermediate host, which suggests its presence also in copepods and humans, hosts from other trophic levels. However, information on human cases, as well as cases from alternative definitive hosts, is limited to sporadic reports. In addition, no studies have investigated the natural occurrence of the tapeworm in its first intermediate host — copepods, as potential host species from this stage were shown only experimentally. All this raises a question on how the life cycle of *D. latus* is maintained in the Alpine region. In this manuscript, we follow the life cycle of *D. latus*, focusing on each host stage, summarising existing knowledge on species range, prevalence rate, and recent history of case reports. We identify knowledge gaps and propose new research directions that would address them. In addition, we discuss several aspects of *D. latus* life cycle that have not been investigated before, such as the transition of *D. latus* eggs into the aquatic environment through sewage, potential alternative definitive hosts, and triploidy in the Alpine population. Considering the life cycle as a whole, we want to emphasise the importance of the multi-faceted approach in studying parasites with complex life cycles. Such an approach can provide better insights into the ecology and epidemiology of *D. latus*, given also that this tapeworm exists in a human-mediated environment. Moreover, we believe that *D. latus* can serve as a model system of parasites with complex life cycles, as well as a model of the effects of polyploidy on host-parasite interactions. Suggesting *D. latus* as a model system goes well in line with the recent calls for large-scale programs of parasite monitoring due to the evident scarcity of information from this ecological group.

## Highlights

• *D. latus*, a tapeworm, requires copepods, fish, and humans to complete the life cycle.
• It is prevalent in fish from Alpine lakes, while human cases are sporadic.
• Natural infection in copepods from the Alpine region remains untested as well.
• Wastewater, alternative hosts, and triploidy are key gaps in transmission.
• We advocate for life-cycle studies across all host stages and transmission routes.

## Graphical abstract

**Figure:**
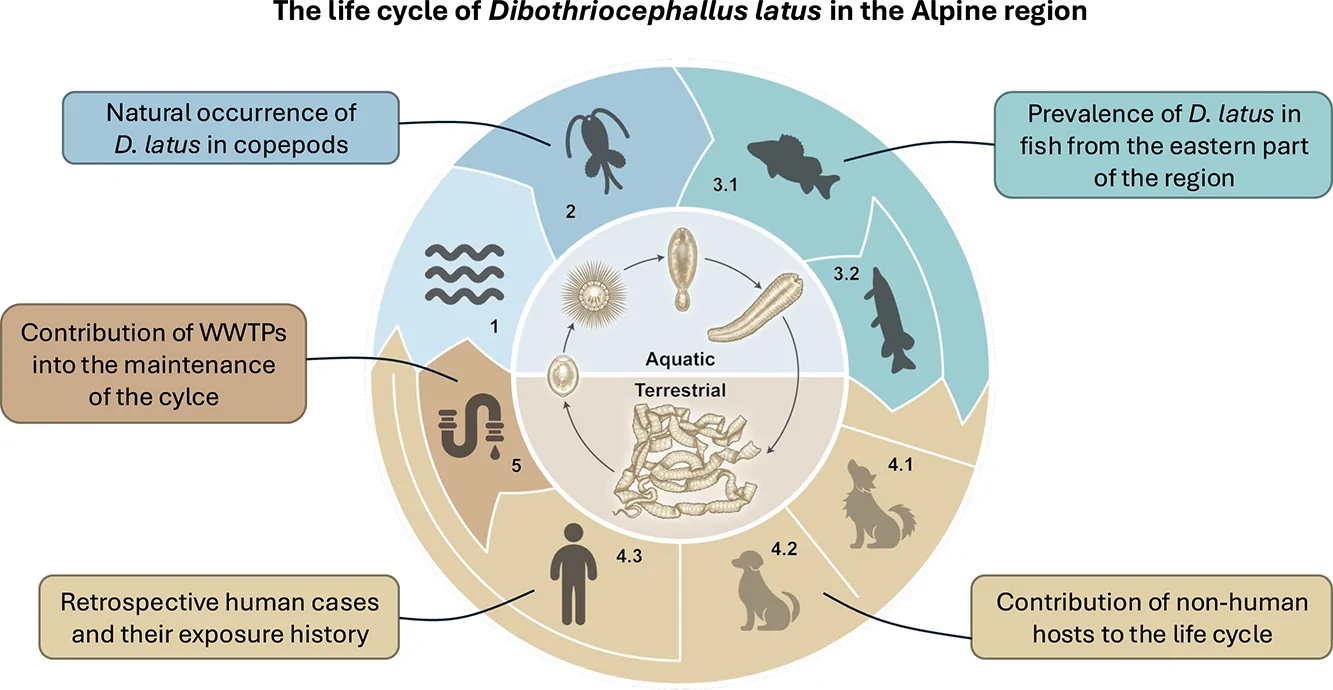

## 1 Introduction

*Dibothriocephalus latus* (Linnaeus, 1758) is the broad fish tapeworm (Cestoda: Diphyllobothriidea), with humans representing its dominant definitive host (Králová-Hromadová et al., 2021). Humans become infected by consuming raw or undercooked fish, a second intermediate host of *D. latus*. Most cases of *D. latus* infections are asymptomatic. However, some infected individuals may experience abdominal pain, diarrhoea, fatigue, and, rarely, pernicious anaemia. Interestingly, while the prevalence of *D. latus* in humans has largely declined over the last 25 years across much of Europe, it remains endemic within Western and Southern parts of the Alpine region (Králová-Hromadová et al., 2021).

Recent studies have shown that plerocercoids, the second larval stage of *D. latus*, are present and abundant in fish from six sub-alpine lakes: Lake Geneva, Lake Neuchâtel, Lake Biel, Lake Maggiore, Lake Iseo, and Lake Como (Gustinelli et al., 2016, Radačovská et al., 2019, Menconi et al., 2020). At the same time, human case reports over the last decade are rather limited. The most recent case of a human infection by *D. latus* was observed in 2023 from Iseo Lake in Italy (Menconi et al., 2025). In Switzerland, human cases re-emerged and started increasing since the 1980s until the 2010s, with the last case recorded in 2010 from Geneva (Dupouy-Camet and Peduzzi, 2004, Wicht et al., 2010a, Králová-Hromadová et al., 2021). However, because of the low pathogenicity and limited medical importance of this parasite, together with the lack of systematic monitoring of cases in Europe, the available data are scarce and probably do not reflect the true prevalence in the population.

*D. latus* has a complex aquatic life cycle with three host stages (Fig. 1). After eggs are released into freshwater (Fig. 1, stage 1), a coracidium, a ciliated motile larva, hatches within 1–2 days. Copepods, the first intermediate host (Fig. 1, stage 2), become infected by ingesting coracidia. After infection, coracidia develop into procercoids in the body cavity of copepods. The second intermediate host is fish (Fig. 1, stages 3.1 & 3.2), which becomes infected with *D. latus* by eating infected copepods or other infected fish. In fish, procercoids develop into plerocercoids. Humans, as well as other fish-eating mammals, serve as definitive hosts (Fig. 1, stage 4), and become infected by consuming raw or undercooked fish. After infecting the definitive host, *D. latus* produces eggs that are released back to fresh water, thus completing the life cycle (Fig. 1, transition from 4.3 to 1).

**Fig. 1:**
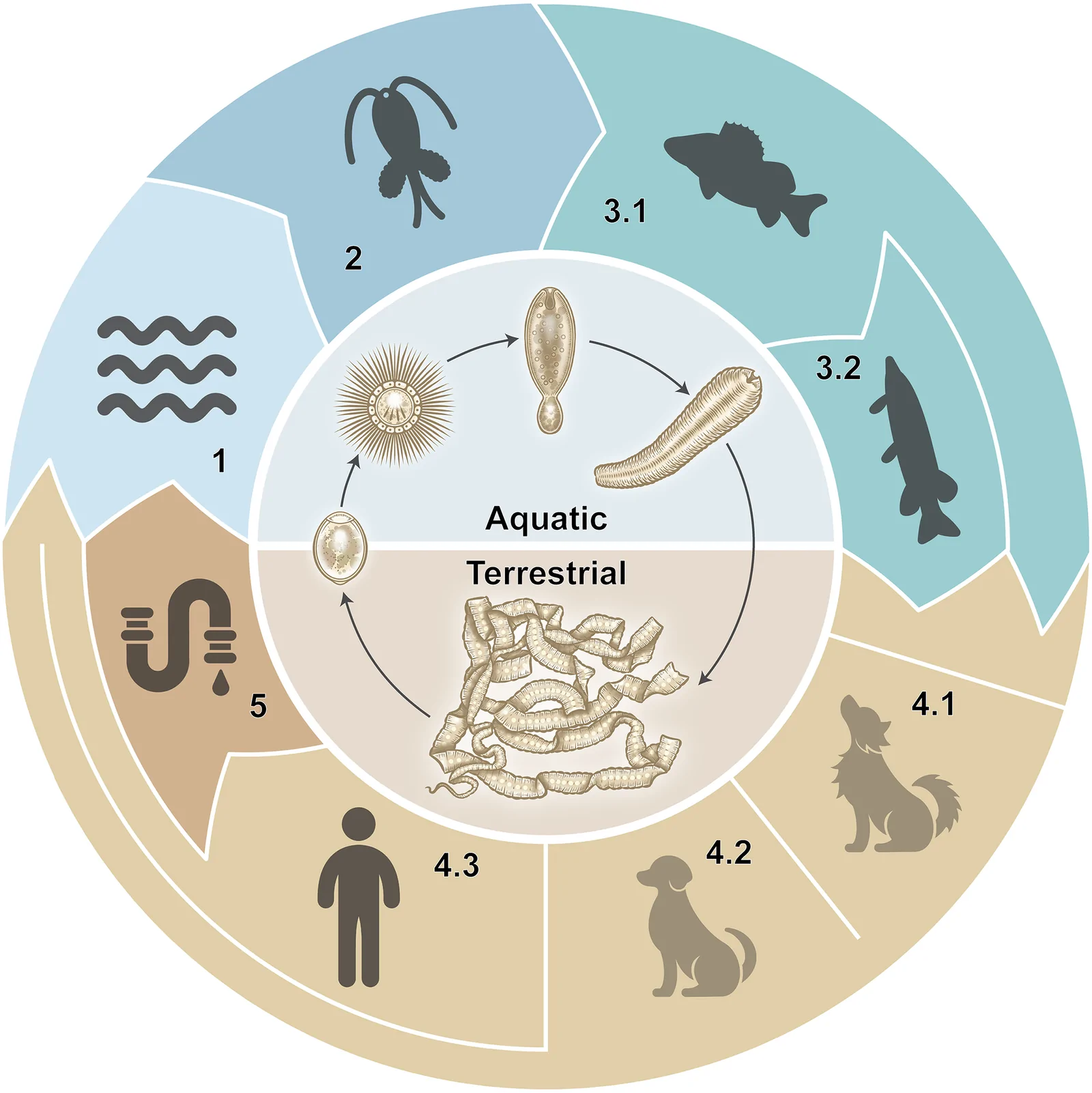
The life cycle of *Dibothriocephalus latus* with indication of different stages. 1 — free-living aquatic stage. 2 — copepods, first intermediate hosts. 3 — fish, second intermediate hosts. 4 — the definitive host. 5 — wastewater treatment plants.

Seemingly simple, thorough observation of this life cycle raises several important questions. First, the notable prevalence of *D. latus* in fish contrasts with sporadic information on prevalence in the definitive host, raising the question about the ecological feasibility of the *D. latus* life cycle maintenance. Second, it is not clear whether *D. latus* is associated only with the French- and Italian-speaking parts of the Alpine region, as no recent information from the German-speaking part is available, neither in fish, nor in humans. Another puzzling aspect of the *D. latus* life cycle is its persistence in urbanised lake systems with substantial wastewater treatment coverage (Fig. 1, stage 5), where parasite transmission is expected to be reduced. Finally, *D. latus* from the Alpine region is found to be exclusively triploid. This triploid form is unique for this area, with a notable exception of lakes in the Patagonia region, where it was brought to from the Alpine region (Torres and Yera, 2018, Orosová et al., 2021, Radačovská et al., 2022b). Such an observation raises further questions on whether triploidy, associated with switching to parthenogenesis, may enable the persistence of *D. latus* in the Alpine region for hundreds of years, either by changing or abandoning its definitive host, or through other evolutionary adaptation.

In this review, we aim to identify and critically revise potential gaps in our understanding of the ecology and epidemiology of this human pathogen by detailing all stages and transitions throughout its life cycle. We will assess the ecological feasibility of the main transmission path (copepods–fish–humans), as well as alternative paths that include other hosts. In addition, we propose directions for further studies that could provide additional information on the life cycle of *D. latus* in a human-mediated environment.

## 2 Life cycle of *Dibothriocephalus latus*

In this section, we follow the life cycle of *D. latus*, concentrating separately on each stage – copepods, fish, and humans – that are supposed to be primary hosts. We provide a short review of available information, such as species range, prevalence rate, and recent history of case reports. At the end of each part, we identify knowledge gaps and formulate questions for further studies.

### 2.1 First intermediate host — copepods

Eggs of *D. latus* are released in freshwater where they develop into a coracidium, a motile ciliated larva, that hatches from an egg within one-two days (Guttowa and Moskwa, 2005). Copepods, the first intermediate hosts, get infected by ingesting coracidia, which than develop into procercoids in a body cavity of copepods. Development in copepods, based on temperature, lasts 16–18 days (Guttowa and Moskwa, 2005).

Copepods were shown to be the first intermediate host of *D. latus* experimentally (Janicki and Rosen, 1917). In this seminal study, several species of copepods from Lake Neuchâtel were exposed to coracidia of *D. latus*. Successful infection and the following entry of the body cavity were observed only in two species — *Cyclops strenuus* and *Eudiaptomus gracilis* (Janicki and Rosen, 1917). Later, copepod species from other areas of *D. latus* distribution (Europe and North America) were tested to detect potential first intermediate hosts. Humes (1950) listed all tested copepod species, categorising them into three groups based on the success of the experimental infection and the development of coracidia. According to this, two copepod species from Europe are supposed to serve as the first intermediate host — *Eudiaptomus vulgaris* and *Eudiaptomus gracilis* (Humes, 1950). Guttowa (1956) suggested that *Eudiaptomus vulgaris* is the primary host, while *Eudiaptomus gracilis* and *Cyclops strenuus* may serve as alternative hosts for *D. latus*. Experimental infection of copepod species from Chile suggested that a potential primary host of *D. latus* in Chilean lakes is *Tumeodiaptomus diabolicus* (Torres et al., 2007).

**Knowledge gaps and questions:** While the first intermediate host was shown experimentally with copepod samples from Lake Geneva and Neuchâtel (Janicki and Rosen, 1917), so far, no studies have explored naturally occurring infection in copepods from the Alpine region. Moreover, copepods were screened for the naturally occurring infection only in Finland Guttowa (1963), where two copepod species – *Cyclops strenuus* and *Thermocyclops oithonoides* – were found to be infected by *D. latus*. At the same time, the need for a copepod screening has been discussed more than 20 years ago by Torres et al. (2004). Observation of naturally occurring infection can support or even expand experimental observations. Screening copepod populations would provide data on actual host species and infection rates. Furthermore, it would shed light on sex- and age-specificity of infection among host species, as experimental studies suggested differences in infection success between age groups and sexes (Guttowa, 1956). In addition, there might be geographic variation in the importance of specific copepod species as the first intermediate host for *D. latus* (Guttowa, 1956).

The presence of susceptible copepod species in a particular lake is one of the prerequisites for the completion of the life cycle. One potential copepod host – *Eudiaptomus gracilis* – inhabits many lakes in the eastern part of the Alpine region (Horváth et al., 2017). This suggests that those lakes are ecologically suitable for *D. latus*, at least in terms of the presence of the first intermediate host. However, observational data on infection rate are required to support this suggestion. Thus, a future direction for studies on *D. latus* life cycle is screening of the copepod species from Swiss lakes to detect natural infection and its geographic distribution.

### 2.2 Second intermediate host — fish

The second intermediate host of *D. latus* is a fish that gets infected by consuming infected copepods. After infection, procercoids attach to internal organs or muscle tissue of fish and develop into plerocercoids. In Europe, four fish species are known to be the most common second intermediate hosts of *D. latus* - perch *Perca fluviatilis*, ruffe *Gymnocephalus cernua*, pike *Esox lucius*, and burbot *Lota lota* (Králová-Hromadová et al., 2021). Perch and ruffe acquire *D. latus* by directly feeding on planktonic copepods. Pike and burbot serve as paratenic hosts as they prey on smaller fish, which can lead to a gradual accumulation of plerocercoids. Several salmonid species in Europe have been reported to be infected by *D. latus* as well (Králová-Hromadová et al., 2021). However, such reports are sporadic, indicating that salmonid species may not represent the primary fish host in Europe.

In Patagonia, in contrast, *D. latus* has been reported in three introduced salmonid species — *Oncorhynchus mykiss*, *Salvelinus fontinalis*, and *Salmo trutta* (Kuchta et al., 2019). Later, *D. latus* has also been detected in several native fish species, such as *Galaxias maculatus* and *Percichthys trucha* (Semenas et al., 2021). Fish surveys in Manitoba, Canada, have found plerocercoids of *D. latus* in three fish species: yellow perch *Perca flavescens*, walleye *Stizostedion vitreum*, and sauger *S. canadense* (Dick et al., 2001). These findings demonstrate that *D. latus*, in the absence of fish hosts common to Europe, can successfully use other fish species as the second intermediate host.

The most recent study on the prevalence of *D. latus* in Switzerland was conducted on perch, including four lakes from the southern and western parts of the country (Radačovská et al., 2020). The reported prevalence was 2% in Neuchâtel Lake (out of 50 examined fish), 6.4% in Geneva Lake (out of 156 fish), 16.7% in Maggiore Lake (out of 48 fish), and 37.5% in Biel Lake (out of 8 fish) (Radačovská et al., 2020). Plerocercoids of *D. latus* were also found in fish from two other lakes in Romandie — Lake Joux in perch and Lake Morat in perch, pike, and burbot (Golay and Mariaux, 1995, Králová-Hromadová et al., 2021).

A review from 1963 on diphyllobothriosis in Switzerland provides a list of *D. latus* findings in fish from lakes and rivers from the German-speaking part of the country (Bouvier et al., 1963), reported mostly at the beginning of the twentieth century. *D. latus* was reported in burbot from Lake Constance, in salmonids from Lakes Thun and Constance (Bouvier et al., 1963), as well as in pike and *Salmo trutta* from Lake Lucerne (Nufer and Nufer, 1905). In addition, *D. latus* was reported in pike from two rivers, the Rhine and the Aare (Bouvier et al., 1963). Reports of *D. latus* from pike and burbot (paratenic hosts) suggest that infection was also present in other fish that get infected directly from copepods. In 1997, a systematic study of parasites in perch from Lake Constance reported no cases of *D. latus* (Balling and Pfeiffer, 1997). However, the authors did not check the muscle tissue, a usual location of *D. latus* plerocercoids in perch. The latest finding of *D. latus* from the German-speaking part of the Alpine region is a plerocercoid sample from perch caught in Lake Brienz, with no clear indication of when this sample was obtained (Yera et al., 2008).

**Knowledge gaps and questions:** Fish species that can serve as a second intermediate host are present in major Alpine lakes. This raises a question about sporadic findings of *D. latus* in the German-speaking part of the Alpine region. More precisely, whether the lack of recent findings indicates that *D. latus* is not present in or has been eradicated from this area, or whether this area was investigated less intensely. Therefore, we propose broader geographic and taxonomic screening of fish for the presence of *D. latus*. Such a screening would target lakes where both potential copepod and fish hosts occur, including as a potential host also salmonid fish as *D. latus* was able to switch fish hosts in Patagonia where it was introduced (Semenas et al., 2021).

### 2.3 Humans as the definitive host

Humans get infected by *D. latus* by consuming raw or undercooked fish. In humans, *D. latus* reaches the adult stage, causing an infection called diphyllobothriosis. Since 1990, a few studies have reported human cases of diphyllobothriosis in France, Italy, and western part of Switzerland. In 1995, Golay and Mariaux (1995) reported the results of a questionnaire administered to hospitals, general practitioners, and private diagnostic laboratories regarding human cases of diphyllobothriosis from the cantons of Fribourg, Neuchâtel, Bern, Vaud, and Geneva in Switzerland. They reported a total of 73 diagnosed cases recorded from 1980 to 1994 (Golay and Mariaux, 1995). Of these 73 diagnoses, it was possible to suggest the origin for 43 cases, with 34 cases classified as of “highly probable” or “possible” local origin (Golay and Mariaux, 1995).

Infection by *D. latus* has been molecularly identified in 20 patients by private physicians in Geneva from November 2004 to August 2006 (Wicht et al., 2007). Of these 20 cases, eight patients reported consuming raw and cooked fish (Wicht et al., 2007). In 2006, seven confirmed cases and one probable case were reported after eating raw marinated perch freshly caught from Lake Geneva (Jackson et al., 2007).

The most recent study reporting human cases of diphyllobothriosis in Switzerland listed eight samples of *D. latus* isolated from infected humans between 2006 and 2008 from the cantons of Geneva, Vaud, Valais, and St Gallen (Wicht et al., 2010a), while the latest human case from the German-speaking part of Switzerland was in St Gallen in 2006 (Wicht et al., 2010a). However, in both cases, there is no information provided on a potential place where the infection happened, locally or in other parts of the Alpine region. Since 2010, no human cases have been reported in Switzerland, which contrasts with the presence of *D. latus* in fish (see above).

In northern Italy, more than 14 cases were reported between 1987 and 2000 (Dupouy-Camet and Peduzzi, 2004). Later, a few more cases were reported from humans (Wicht et al., 2010a, Wicht et al., 2010b). The last human case reported from Italy origins from Lake Iseo (Menconi et al., 2025). In France, over 70 human cases were reported between 1993 and 2002 (Dupouy-Camet and Peduzzi, 2004) with no public reports on human cases since then.

**Knowledge gaps and questions:** The lack of reported cases may not imply a lack of human infections, but simply a lack of reporting, as there have been no studies focused on *D. latus* infections in humans since 2010. This may suggest that, if human cases happen, they remain unreported. Nevertheless, results of the questionnaire conducted by Golay and Mariaux (1995) revealed that hospitals, general practitioners, and private laboratories collected data on diphyllobothriosis cases, showing on average 13 cases per year from 1990 to 1994 (Golay and Mariaux, 1995). Thus, contacting medical practitioners may provide data on more recent cases of diphyllobothriosis and their dietary drivers. Covering different parts of the Alpine region may also reveal the geographic distribution of those cases, indicating whether *D. latus* is indeed associated only with French- and Italian-speaking parts of the region. Such an approach may also help to understand the status of *D. latus* in Czech Republic, where a recent report on a human autochthonous case contrasts with the absence of infection in fish (Scholz et al., 2024).

The very reappearance of human cases of *D. latus* (or their reports) in the Alpine region was linked with increased popularity of raw fish consumption (Dupouy-Camet and Peduzzi, 2004). At the same time, it is not clear whether increased raw fish consumption has a regional character, as was suggested by Dupouy-Camet and Peduzzi (2004), to explain geographic variation of human cases. Data on cantonal fish consumption or availability of restaurants serving locally caught fish nearby lakes may indicate whether there is a geographic difference. Recent increase in raw fish consumption is highly likely to have contributed to the maintenance of the *D. latus* life cycle. Such changes in feeding behaviour, however, may not be able to explain how the cycle was maintained before. *D. latus* was introduced to South America at the end of 19th century, suggestively from the Alpine region (Radačovská et al., 2022b). This can indicate that *D. latus* should have been present in Alpine lakes for more than 130 years in already triploid form.

## 3 Wastewater, other definitive hosts, and triploidy

In this section, we address topics connected to the life cycle of *D. latus*, that, while being mentioned before, received less attention historically. First, we discuss the transfer of *D. latus* eggs from humans into the aquatic environment, especially through wastewater treatment plants (WWTPs), given that *D. latus* persists in urbanised environments. Second, we investigate the possibility of *D. latus* to use other piscivorous mammals as a definitive host, given the lack of reports on human cases. Finally, we focus on the interesting case of triploidy in the Alpine region, and discuss whether ploidy has any effects on the life cycle and host-parasite interaction.

### 3.1 Wastewater treatment plants

When *D. latus* reaches maturity in the definitive host, it starts producing eggs. To complete the cycle, eggs must end up in an aquatic environment. Up to 97.3% of Swiss residents were connected to the system of WWTPs in 2024 (FOEN 2024 Indicator WS076), suggesting that eggs of *D. latus* reach the aquatic environment passing through sewage. This, however, raises the question whether eggs of *D. latus* can pass through WWTPs.

**Egg survival:** A systematic review on dispersal of *Taenia* spp. eggs from several countries has shown that WWTPs do not fully remove helminth eggs from water (Jansen et al., 2021). In addition, *Taenia* eggs were able to survive in anaerobic sludge (Jansen et al., 2021). Comparison with *Taenia* spp. may, however, not hold as species from this genus do not require an aquatic stage, while eggs of *D. latus* hatch in water, which may reduce their survivability passing through WWTPs. A few studies from *D. latus* population in Russia have shown that eggs can survive in septic tanks (Bizyulyavichus, 1968) and can be detected after passing through WWTPs, even after the UV treatment (Tonoeva et al., 2018). The latter study, however, did not check the viability of eggs passed through WWTP, which may depend (together with number of eggs filtered out) on the WWTP type. In case of Switzerland, not all WWTPs have a stage with sand filtration that can potentially filter out eggs of *D. latus*. For example, on the Swiss side of Lake Geneva, 61% of the population has access to WWTPs without sand filtration out of the total population of 1’950’000 connected to WWTPs (of Topography swisstopo, 2026). Alternatively, the cycle of *D. latus* can be completed, avoiding WWTPs, either due to overflow events at WWTPs or due to direct faecal contamination of lakes.

**Overflows:** An alternative way for eggs to reach lakes is due to overflow events at WWTPs (Gustinelli et al., 2016, Menconi et al., 2021). One study from Lake Iseo in Italy showed that the prevalence of *D. latus* in perch correlated with concentration of *E. coli*, a parameter that serves as an indicator of faecal contamination of water (Menconi et al., 2021). Both parameters were linked to the proximity to WWTP. This suggests bypass events at the local WWTP, leading to contamination of the water with untreated sewage. Publicly available data on bypass events and their volume in Switzerland is limited to a few cantons and cities. For example, in Canton Vaud, the total volume of bypass water in 2024 was around 3.2% (de Vaud, 2025). In Lausanne only, the total volume of bypassed water was around 7.5% in 2024 (S.A., 2025). Apart from limited geographic coverage, there is no information on the seasonality of overflow events. Such data can be useful to compare with seasonal variation of *D. latus* prevalence in copepods.

**Fishermen:** It was hypothesised that the presence of infected fish indicates that the life cycle is completed due to faecal contamination of lakes by professional and leisure fishing (Dupouy-Camet and Peduzzi, 2004). Existing reports on human infection contain no data on the professional occupation of patients and whether they have any connection with fishing activities. To test this assumption, it would require collecting occupational data during contact with medical practitioners.

**Human reservoirs:** Irrespective of how eggs reach lakes, it is important to estimate how many eggs do so to better understand the epidemiological dynamics. Studies indicate that one parasitic worm has a high reproductive output, producing around one million eggs daily (Králová-Hromadová et al., 2010). Given that diphyllobothriosis can develop without symptoms, some amount of eggs can be released before *D. latus* is detected and treated. Thus, the question is how many people would be required to sustain a population of *D. latus* in the Alpine lakes? Even a high (but not complete) egg removal rate can still result in a significant number of eggs reaching the aquatic environment, hatching and infecting the first intermediate host. The geographical location of WWTPs may matter as well. For example, the population of *D. latus* in Lake Geneva may receive the inflow of eggs from Lausanne, where a WWTP is located on the lake, but not from the city of Geneva, where WWTPs are located on the river Rhône down the lake. Thus, we propose a molecular screening of wastewater to identify and map locations where *D. latus* eggs enter the aquatic environment. For example, testing influent water at WWTPs can indicate the presence of *D. latus* in the sewage system. While testing effluent water at WWTPs can indicate whether biological material passes through.

### 3.2 Other animals as the definitive host

The life cycle of *D. latus* requires a mammal as a definitive host to complete the cycle. Humans are supposed to be the primary definitive host. However, other mammalian species consuming fish can also contribute to sustaining the life cycle (Králová-Hromadová et al., 2021). Below, we discuss the existing literature on the prevalence of *D. latus* in other mammals, separating wild from domestic animals, since the latter may have higher exposure to fish infected with *D. latus* due to contact with humans.

**Domestic animals:** Among domestic animals, dogs are supposed to be the most suitable hosts (Králová-Hromadová et al., 2021). Their significance, however, may vary regionally. For example, in the Andean Patagonia, dogs are likely to be one of the most important definitive hosts together with humans (Semenas et al., 2021). Analysis of faecal samples of free-roaming urban dogs in Bariloche, Argentina, showed that 20 out of 118 faeces were positive for *D. latus* (Roth et al., 2018). It contrasts with results of coprological studies on dogs from Switzerland, where *D. latus* was detected only in two dogs out of 505 in 2006 (Sager et al., 2006) and only in one dog from Neuchâtel out of 2065 tested in 2025 (Schneider et al., 2025). It was reported that the dog from Neuchâtel belonged to a fisherman and was repeatedly fed raw fish freshly caught from Lake Neuchâtel (Schneider et al., 2025). A similar low prevalence was observed among cats in Switzerland, where *D. latus* was detected only in a faecal sample from a single cat out of 664 tested (Zottler et al., 2019). This suggests that the contribution of domestic animals to the life cycle of *D. latus*, at least in Switzerland, is limited.

**Wild animals:** Among wild animals, wolves, foxes, and lynx are mentioned to be the most commonly reported hosts of *D. latus* (Králová-Hromadová et al., 2021). Similar to domestic dogs, there seems to be a regional variation in how significant each of these species is for the life cycle of *D. latus*. For example, an analysis of 19 faecal probes of wolves in the Białowieża forest (Poland) found that six of them (31.6%) had *D. latus* eggs (Górski et al., 2006). Researchers explained this prevalence with pollution of the local river with pesticides, after which many fish were lying dead on the riverbank (Górski et al., 2006). At the same time, this finding should be treated with a caution as the study identified the species based on eggs morphology only, which could be misleading due to similarity of *D. latus* eggs to species from the *Spirometra* genus. In comparison, a recent study from Switzerland analysed post-mortem samples from 121 wolves across different parts of the country and found no cases of *D. latus* (Schneider et al., 2025).

There is only one report of *D. latus* in foxes in the Alpine region from 2010 (Wicht et al., 2010a). The location, however, is interesting, as this sample of *D. latus* originates from the canton of Grisons, which is located in the eastern part of Switzerland, far from major lakes. In addition, one potential case of *D. latus* has been mentioned in a study investigating intestinal parasites of foxes from the canton of Geneva (Reperant et al., 2007). In the study, 228 foxes were examined post-mortem from 1998 to 2002, with only a single recovered specimen of “*Diphyllobothrium spp.*” (Reperant et al., 2007). Another carnivorous species, the lynx, was assumed to be a host of *D. latus*, for example, in Poland (Szczęsna et al., 2008). However, morphological identification was not confirmed molecularly. In addition, the most recent study on post-mortem examination of 346 lynx reported no *D. latus* for the period 2000–2022 in Switzerland, suggesting that lynx is an unlikely host in this area (Borel et al., 2026).

Otters could also serve as a potential host for *D. latus* in the Alpine region. They reappeared in Switzerland in 2009, and several observations were made near Lake Geneva (Angst and Weinberger, 2020). Otters, compared to the other animals mentioned above, can actively hunt fish and are known to feed on perch (Prigioni et al., 2009), the second intermediate host of *D. latus*, which, while not confirmed before, makes them a potential target of infection. Moreover, such infection has been observed in Poland, where around 2% of investigated otter faeces tested positive for *D. latus* (Górski et al., 2006, Górski et al., 2010). At the same time, given their time of reappearance, otters likely did not contribute to the life cycle prior to 2009.

### 3.3 Triploidy and its effect on *D. latus*

All the analysed samples of *D. latus* from the Alpine region (128 samples) were found to be triploid in contrast with exclusively diploid samples from Russia and Finland (Wikgren and Gustafsson, 1965, Radačovská et al., 2022a). Triploidy was also detected in all *D. latus* samples from Argentina, which showed relatedness to the Alpine population (Radačovská et al., 2022b). While triploidy has been observed among main groups of parasitic worms (Kovalov and Trubenová, 2026), its ecological or evolutionary consequences remain unclear. Thus, triploid populations of *D. latus* can serve as a model system to investigate potential effects of triploidy, for example, on host-parasite interaction. It was shown that triploid trematodes develop faster and reach larger body size compared to diploid ones (Blair, 2022), potentially increasing parasite virulence. Does this pattern hold in case of triploid *D. latus*? If yes, would this affect intermediate host mortality and transmission rate?

Another question with triploid population of *D. latus* is the time when triploidy appeared in the Alpine region. First documented cases of *D. latus* in Switzerland date back to the end of the 16th century (Radačovská et al., 2022a). Patagonian triploid population of *D. latus* is suggested to appear at the end of the 19th century (Semenas et al., 2021). While Patagonian and Alpine populations are closely related (Radačovská et al., 2022b), it is not clear whether triploidy has appeared first in the Alpine region and then was brought to Patagonia, or appeared independently in both regions. In the first case, one can assume that triploidy appeared in the Alpine region at least 130 years ago. However, dating the approximate origin of triploidy would require a separate analysis, including also historical samples. In addition, several human cases of *D. latus* in Patagonia, that originate from Eastern Europe, may not be triploid (Semenas et al., 2024). Thus, sampling *D. latus* from other regions with the following ploidy assessment may provide more data, as the ploidy level was investigated only in the Alpine region and from several locations in Patagonia and Russia.

The triploid population of *D. latus* is proposed to reproduce parthenogenetically, as samples from the Alpine region showed abnormal spermatogenesis (Orosová et al., 2021). This would suggest that all individuals reproduce clonally without recombination. Parthenogenetic lineages are often considered evolutionary dead ends due to the accumulation of deleterious mutations. Given the suggested lack of outcrossing, would it be possible to estimate mutational load comparing different triploid lineages of *D. latus* in the Alpine region? In addition, asexuality seems especially paradoxical among parasitic animals, as they experience constant selective pressure from their hosts. However, asexual parasitic animals tend to have broader host ranges (Gibson, 2019), which is potentially the case of *D. latus* as it uses other species of copepods and fish in South America (see above). Thus, the question arises whether triploidy and/or asexuality allowed *D. latus* to expand its host range. Should one also expect alternative hosts in the Alpine region?

## 4 Concluding remarks

*D. latus* is a parasite with a complex life cycle that requires not only the presence of three suitable hosts, but also a trophic connection between them. Notable presence of *D. latus* in fish in Swiss lakes indicates that some fraction of the suitable first intermediate host, as well as the definitive host, should be infected as well, for the life cycle to be completed. However, existing literature is rather limited in terms of providing a clear image of how the life cycle of *D. latus* is maintained. In this paper, we reviewed existing knowledge on *D. latus*, concentrating on each stage and proposing further research directions.

The very focus on the life cycle of *D. latus*, not on particular stages, requires a multi-faceted approach. Screening planktonic copepods would provide data on the natural infection rate, as well as support or expand experimental studies of suitable first intermediate hosts. Data from parasitological monitoring of fish from different lakes may help to identify alternative fish hosts. Data from medical practitioners and diagnostic laboratories is needed to obtain hidden statistics on human infections and their origin. This should be accompanied by parasitological investigations of other piscivorous mammals to detect their role in the life cycle maintenance. Apart from this, one should consider transitions between hosts as well. Given that *D. latus* is a food-borne parasite, studying culinary practises from different parts of the Alpine region can reveal regional differences in fish consumption and, thus, infection probability. Because of the high coverage of WWTPs, it is also important to investigate how eggs pass through the sewage system.

Such a multi-faceted approach can make *D. latus* a potential model system for investigating tapeworms with complex life cycles. Including all hosts with trophic connections between them, as well as expanding the temporal and geographic scope of research, should provide better insights into the ecology of such systems. Better understanding of the ecology can also reveal important epidemiological aspects of *D. latus* transmission, as well as more general transmission dynamics of fish-borne zoonotic parasites. Moreover, *D. latus* can serve as a model system to study the effect of ploidy on the host-parasite dynamics as *D. latus* exists in two ploidy forms (Kovalov and Trubenová, 2026). All this goes in line with a recent study calling for large-scale parasite monitoring programs (Hammoud et al., 2026), as our understanding of long-term trends in parasite systems is still limited due to data scarcity.

## CRediT authorship contribution statement

**Viktor Kovalov:** Writing – review & editing, Writing – original draft, Investigation, Conceptualization. **Barbora Trubenová:** Writing – review & editing, Writing – original draft, Conceptualization.

## Ethical statement

Non-applicable

## Financial support

This work has been supported by the 10.13039/501100001711Swiss National Science Foundation Starting Grant TMSGI3_218475: *Drug resistance evolution across taxonomic boundaries: investigating the effects of ploidy, lifestyle, and reproductive strategy*.

## Declaration of competing interest

The authors declare that they have no known competing financial interests or personal relationships that could have appeared to influence the work reported in this paper.
